# Role of medical and molecular imaging in COPD

**DOI:** 10.1186/s40169-019-0231-z

**Published:** 2019-04-15

**Authors:** Lukasz A. Myc, Yun M. Shim, Victor E. Laubach, Julien Dimastromatteo

**Affiliations:** 1 Department of Medicine, Division of Pulmonary and Critical Care Medicine, University of Virginia School of Medicine, P.O. Box 400546, Charlottesville, VA USA; 2 Department of Surgery, Division of Thoracic Surgery, University of Virginia School of Medicine, P.O. Box 801359, Charlottesville, VA USA; 3 Department of Biomedical Engineering, University of Virginia School of Medicine, P.O. Box 800759, Charlottesville, VA 22908 USA

## Abstract

Chronic obstructive pulmonary disease (COPD) is expected to climb on the podium of the leading causes of mortality worldwide in the upcoming decade. Clinical diagnosis of COPD has classically relied upon detecting irreversible airflow obstruction on pulmonary function testing as a global assessment of pulmonary physiology. However, the outcome is still not favorable to decrease mortality due to COPD. Progress made in both medical and molecular imaging fields are beginning to offer additional tools to address this clinical problem. This review aims to describe medical and molecular imaging modalities used to diagnose COPD and to select patients for appropriate treatments and to monitor response to therapy.

## Background

Chronic obstructive pulmonary disease (COPD) remains a leading cause of mortality worldwide [1]. Clinical diagnosis of COPD has classically relied upon detecting irreversible airflow obstruction on pulmonary function testing (PFT) as a global assessment of pulmonary physiology. The natural history of COPD is characterized by an irreversibly progressive decline in lung function, with the pathophysiology resulting in airway obstruction characterized by either the loss alveolar gas exchange units and elasticity or the development of muco-fibrotic airway remodeling. These paradigms have served as the basis for the two classic COPD phenotypes: emphysema and chronic bronchitis. However, this basic dogma has been challenged in recent years as varying degrees of co-existing emphysema, chronic bronchitis, and potentially significant vascular pathologies have been appreciated in patients with COPD [2–6].

While sharing the common terminal phenotype of irreversible airflow limitation, emphysema and chronic bronchitis are fundamentally different diseases and are suspected to have unique biomolecular mechanisms underpinning their pathogenesis. While PFT is a highly sensitive and relevant modality to detect airflow limitations and gas exchange defects, it lacks regional sensitivity and anatomic specificity to refine phenotyping of COPD.

Currently, chest X-ray, CT and magnetic resonance imaging (MRI) are the most matured imaging modalities to phenotype COPD. These modalities are non-invasive, feature high resolution images based on different physical properties and provide anatomic detail (Table 1). Similar to chest X-ray, CT uses radiographic X-ray technology and is based on attenuation of X-rays that occur after absorption and scattering through the tissue. Denser tissues such as bone attenuate signal to a greater extent than less dense tissues such as lung [3, 7]. The loss of lung tissue and elasticity in emphysema can be exploited to determine presence or absence of emphysematous lung by pixel-by-pixel quantification of these signals emanating from lung tissues [3]. Application of CT techniques to characterize airway disease, however, has been more challenging due to lack of correlations between anatomic airway alterations detectable by CT and pathologic airflow limitation. MRI employs magnetic resonance of hydrogen protons to generate images. As body content is mainly fluid (H_2_O), MRI provides contrast between tissues with varying degrees of fluid content. In addition, hyperpolarized gases used as contrast agents can be employed to image the lung with MRI.

**Table 1:** Imaging modalities general characteristics

Modality	Physical principle	Spatial resolution	Parameters imaged	Depth	Acquisition time	Cost per scan^a^ (US $)
PET	Radioactivity	1–2 mm	Metabolism, immunology	No limit	minutes/hours	$1000–$2000
SPECT	Radioactivity	1–2 mm	Metabolism, immunology	No limit	minutes/hours	$1000–$1500
MRI	Electro-magnetism	< 100 µm	Structure, metabolism	No limit	minutes/hours	$800–$1200
CT	X-ray	< 100 µm	Structure	No limit	seconds	$600–$800

^a^Cost per scan may vary depending on the injected dose price. Adapted from [52]

Conventional imaging methods can be augmented by using molecular imaging methods such as single photon emission computed tomography (SPECT) and positron emission tomography (PET) with the purpose of characterizing physiologic derangements associated with COPD with greater resolution. Molecular imaging becomes feasible with the administration of radioactive isotopes conjugated to molecules (i.e. radioligand) that are designed to specifically bind to molecular targets in tissue that are diagnostic of a pathology. SPECT and PET modalities feature high sensitivity and provide molecular and metabolic detail regarding the tissues of interest. Such detailed information may allow discrimination between two group of patients with unique molecular or cellular profiles. The physical principle behind SPECT is based upon the detection of radioactive emission of gamma photons from specific isotopes such as ^99m^Tc, ^123^I or ^111^In. In simplest terms, the energy of gamma photons, measured in kilo electron Volt (keV), is high enough to pass through any type of tissue and reach the detector after collimation. The technology behind PET scanning varies slightly from SPECT as it retains radioactivity as the key phenomenon. PET imaging is based on the collision of positrons emitting from the isotope (such as ^18^F, ^64^Cu, ^68^Ga or ^89^Zr) nucleus with orbital electrons. The collision leads to the generation of two photons of high energy 511 keV emitted in strictly opposite directions. This geometrical characteristic is a key feature of PET imaging as it allows deconvolution of the signal acquired by the detector and thus localization of the emitting molecule. The event captured by the detector will be taken into consideration only if two photons hit the detector at the same time and at 180° from each other. For more information about the physics-based principles underpinning medical imaging, we direct the reader to the textbook *The Essential Physics of Medical Imaging* [8].

Currently, molecular imaging is not a first-line method for the diagnosis of COPD. However, it can provide information for enhancing diagnosis, advancing individualized disease-phenotyping, and assisting with the assessment of responses to treatments. In this review, we will focus on how conventional medical imaging is used in COPD diagnosis, disease assessment/prognosis, treatment selection, and therapeutic development as well as how emerging techniques of molecular imaging can augment these conventional tools and possibly improve patient-centered outcomes.

## Imaging modalities in disease assessment

Moving beyond the simply descriptive utility, imaging modalities have the potential to provide new opportunities to advance research and clinical care for patients with COPD. Imaging modalities can often accurately characterize the pathology underlying COPD. As powerful technologies, imaging modalities can grade disease severity, prognosticate, and personalize phenotyping of COPD. Therefore, exploiting appropriate imaging modalities and metrics may enhance selection of “right patients” for “appropriate” therapies in prospective clinical trials. Such gains can allow for leaner and shorter clinical trial designs that employ imaging endpoints as surrogates for efficacy while retaining generalizability. Development of COPD takes several decades, and this is a major barrier to conduct clinical trials to assess effects of new therapies within a reasonable duration (i.e. 6–18 months). This last point is worth emphasizing as the conventional and emerging imaging technologies can help overcome these challenges.

While the landscape of current imaging methods may be complex, it can be approached in a simplified anatomic scheme where COPD can be seen as affecting one or more of the following compartments: (1) conducting airways (bronchi and bronchioles), (2) airspaces (alveoli) and interstitium involved in gas exchange, or (3) pulmonary vasculature. Perturbation of pulmonary physiology in these compartments can change the functions of ventilation, gas exchange and/or perfusion. Imaging modalities can then be divided into those that assess the anatomy (static biometrics) and those that assess the function of the anatomy (dynamic biometrics).

The following sections provide brief overviews of the various available imaging techniques and the quantitative methods that have been developed on the basis of these modalities. We will then discuss static and dynamic imaging markers that have been employed in prognostication and population selection for therapy and as clinical trial endpoints in COPD.

### Computed tomography

CT has been the most developed and advanced imaging modality in COPD. While CT imaging has certainly surpassed traditional chest X-ray in allowing for the detection of mild forms of emphysema, CT also allows for precise and quantitative analysis of pathologic patterns while simultaneously providing information regarding regional severity of disease, which is a significant advantage when compared with the global, low resolution data furnished by PFT.

Anatomically considered, airways, airspaces and vasculature have all been evaluated using CT, and a variety of biometrics have been explored. The most widely adopted CT applications have been those developed for quantification of airway thickness and the extent of emphysema. However, methods to quantify functional air trapping as well as vascular pathology have also been investigated.

**Fig. 1:**
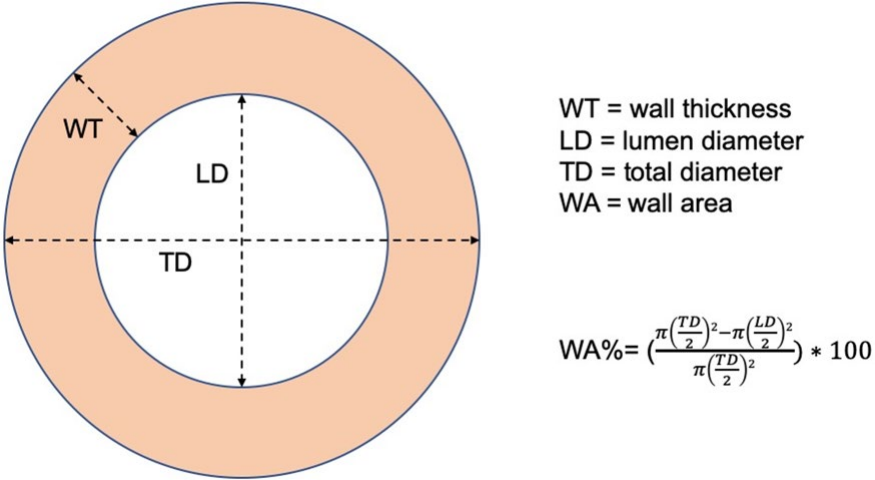
Bronchial anatomy measurement explanatory diagram

Airway thickness is thought to reflect either the extent of airway inflammation or perichondral fibrosis and has been assessed using a variety of indices which have included: (1) airway wall area (WA), defined as the luminal area in cross section subtracted from the area of an airway in cross section where the radius of the circle extends to the outer edge of the airway wall (A_o_), (2) ratio of airway thickness to total diameter of the airway, (3) percentage wall area (WA%), defined as WA/A_o_ x 100 [3, 7] (Fig. 1), and (4) Pi10, which predicts the square root of the wall area for a hypothetical airway with an internal luminal perimeter of 10 mm. Importantly, WA% has been demonstrated to be inversely correlated to spirometric markers of obstruction such as FEV1 percent predicted [7].

Both qualitative and quantitative CT evaluation of emphysematous disease has been undertaken. While qualitative radiologic assessment of emphysema and distribution have been used in clinical trials, low attenuation area percent (LAA%, defined as the % of voxels below a certain threshold attenuation at total lung capacity, most often − 950 HU but also − 910 HU and − 960 HU) has become a standard value used to characterize emphysema extent and has demonstrated good inverse correlation with diffusing capacity and FEV1 [9].

Pulmonary air trapping has been assessed using both static and dynamic CT applications. For example, the ratio of mean lung density (MLD) on expiratory imaging referenced to MLD on inspiratory imaging was demonstrated to correlate with FEV1 percentage predicted and the ratio of residual volume to total lung capacity (RV/TLC); a plethysmographic measure of air trapping. Additionally, LAA% inferior to − 856 HU on expiratory CT has been employed as a static measure of air trapping [10].

Vascular pathology has not been well elucidated in COPD but is thought to involve distal pruning of blood vessels, endothelial dysfunction and a lack of inhibition of the hypoxic vasoconstrictive response in the presence of inflammation. As a global static measure, the pulmonary artery-to-aorta (PA/Ao) ratio is readily obtainable by CT and believed to reflect the downstream pressure of the pulmonary arterial tree. As a surrogate for increased pulmonary pressure, PA/Ao outperformed echocardiography and was reported to linearly correlate with mean PA pressure in patients with COPD [5]. A more advanced but time intensive procedure has been the attempt to quantify the heterogeneity of regional lung perfusion. For instance, MDCT-based assessment of pulmonary blood flow has also been used to evaluate heterogeneity of pulmonary perfusion in patients with subclinical emphysema and reported an increased coefficient of variance of both PBF and mean transit time [11]. Similarly, Iyer et al. [5] reported on the use of dual energy CT to assess pulmonary blood volume and its coefficient of variance, presumed surrogates of pulmonary perfusion and its distribution. Finally, segmentation of the pulmonary vasculature has allowed for the measurement of the volume of vessels less than 5 mm^3^ (BV5) standardized to the total blood vessel volume as well as the non-vascular tissue volume (surrogate for V/Q mismatch), the latter of which was reported to be inversely related to pulmonary function test, diffusion capacity of carbon monoxide (DLCO) [12]. Considered together, vascular biometrics have correlated with exacerbation rates, response to vasodilators and resting oxygen saturation.

### Magnetic resonance imaging

#### Noble gas MRI

One of the most compelling motives to adopt MR imaging in the evaluation of pulmonary pathology has been the reality that this modality obviates the risks associated with ionizing radiation. While in previous decades MR imaging of the lung was greatly hindered due to the multiple air-tissue interfaces that resulted in magnetic field artifacts and poor signal-to-noise ratio, the advent of hyperpolarized gas (e.g. ^3^He) MRI has significantly advanced this field. Similar to CT, MRI is able to provide qualitative and quantitative regional data not otherwise obtainable by PFT. Early hyperpolarized gas MRI was performed with helium gas due to its greater magnetic moment which offered good image resolution. However, relative scarcity of ^3^He as well as the ability for higher levels of polarization has resulted in the ascendancy of hyperpolarized ^129^Xe MRI imaging. To date, ventilation defect percentage (VDP) and apparent diffusion coefficient (ADC) have been the principal parameters acquired with lung MRI. ADC is believed to reflect the theoretical Brownian motion of gas particles in the airspaces and is considered particularly sensitive in the detection of microstructural disease (e.g. very mild emphysema) [13]. Not only has ^3^He ADC correlated well with DLCO, it has demonstrated the capacity to distinguish COPD patients from healthy controls [14].

**Fig. 2:**
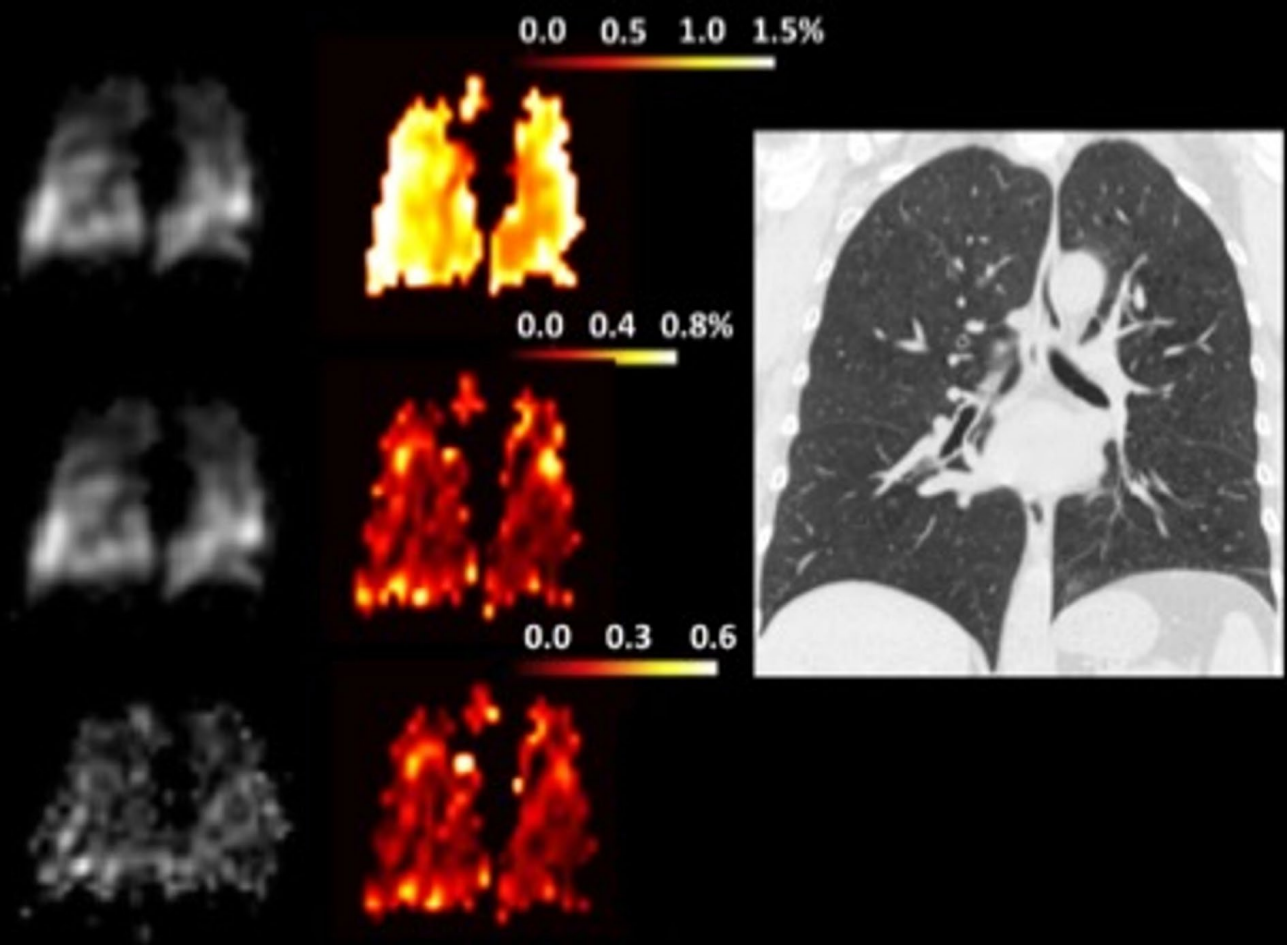
High resolution CT and hyperpolarized ^129^Xe dissolved-phase MR images in patient with COPD. In order, image reconstructions of gas (top left), tissue (left middle) and red blood cells (bottom left) compartments are presented. The paired color gradient images are fused reconstructions demonstrating relative gas content in the different compartment; tissue/gas, RBC/gas and RBC/tissue. Adapted from [16] with permission

Aside from its relative abundance, hyperpolarized ^129^Xe imaging has the incremental benefit over ^3^He in that it is highly tissue-soluble and possesses unique MR spectral peaks enabling the identification of different physical compartments, specifically ^129^Xe dissolved in the lung parenchyma and blood (Fig. 2) [15, 16]. Although the feasibility of employing hyperpolarized ^129^Xe MR to simultaneously image the distribution of both ventilation and gas uptake in COPD has been reported, to date no published studies have identified unique metrics relevant to specific phenotypes in COPD.

#### Oxygen-enhanced MRI

In parallel with the development of hyperpolarized noble gas methods, research employing oxygen-enhanced pulmonary MRI (OEMRI) for functional lung imaging has also evolved. Oxygen-enhanced MRI (performed with subjects breathing 100% oxygen) relies on the weakly paramagnetic character of molecular oxygen and so relies on a T1 effect, allowing the use of conventional proton imaging (and so MR scanners) without the need for specialized radiofrequency coils [17]. This cost-sparing feature of OEMRI together with its capacity to assay both regional ventilation and oxygen transfer make it a compelling candidate for routine functional pulmonary imaging [18]. It remains to be seen whether the incremental information available with hyperpolarized noble gas methods is worth the additional costs associated with those technologies.

### Nuclear medicine imaging

#### Positron emission tomography

Early detection of COPD can potentially afford more therapeutic opportunities for patients. Numerous cellular and molecular events are presumed to occur distinctively during the early or late phases and can reflect signatures that can be targeted as the readouts of the imaging modalities. The current trend in the molecular imaging field is to develop these tools that can accurately identify and quantify the cellular and molecular derangements involved in the pathogenesis of COPD.

Most patients with COPD are diagnosed at advanced stages, and the biomolecular characteristics of these types of COPD patients are well established [19–25]. A number of molecular imaging tools have been developed to assess the inflammatory responses at the cellular level in varying stages of COPD pathogenesis.

**Fig. 3:**
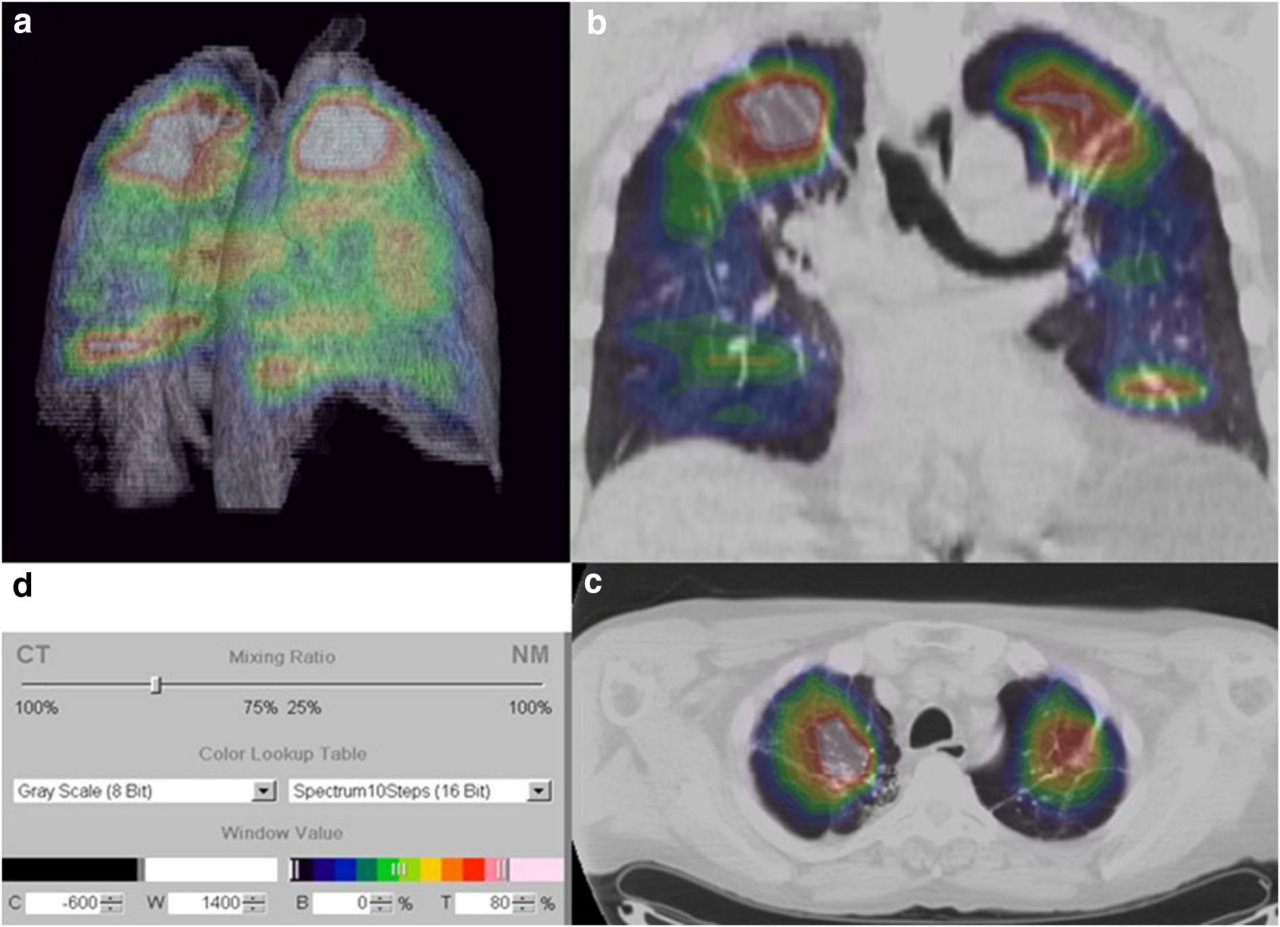
**a** Three-dimensional, **b** axial, and **c** coronal imaging illustrating the predominantly apical distribution of pulmonary ^18^fluorodeoxyglucose ([^18^F]-FDG) uptake in a patient with usual chronic obstructive pulmonary disease (COPD). **d** The color scale gives the spectrum applied to the full range of [^18^F]-FDG uptake, so that each color band represents a range of 10% of the maximum signal, with the maximum signal represented by *white* and the minimum signal by *black*. (Reprinted from [28] with permission of the American Thoracic Society. Copyright © 2017 American Thoracic Society.)

^18^F-fluorodeoxyglucose (FDG) is the most commonly used PET radiotracer in nuclear medicine. FDG is an analog of glucose that becomes trapped in glucose-avid cells. Increased FDG accumulation in cells thus reflects increased glucose metabolic activity. Consequently, a positive signal on FDG PET images is the result of contrast between cells or tissue with high glucose metabolism and those with low glucose metabolism. FDG has been used to detect pathology characterized by active inflammation. As applied to lung pathology, Jones et al. [26] demonstrated a relation between FDG uptake and activated neutrophils. However, it is important to remember that FDG PET is not a direct indicator of neutrophils but reflects relative glucose metabolism. As the role of inflammatory cells such as neutrophils has been well established in the case of COPD and asthma, Jones et al. [27] highlighted the potential of using FDG in addition to the use of a macrophage-targeted ^11^C-PK1195 PET radiotracer to discriminate asthma from COPD in patients. Subsequently, a study of 30 patients divided into healthy, alpha1-antitrypsin deficiency and COPD groups reflecting different severities of inflammation, showed that FDG PET could differentiate these three study groups (Fig. 3) [28]. Additionally, FDG uptake was correlated with the severity of COPD as assessed by FEV1 measurement. Despite these interesting results, quantification of FDG uptake by PET remains a challenge due to differences in tissue composition (parenchymal/airway and endothelial cells) and immune cells, air, blood and water content. Methods of improved image quantitation have been developed to accommodate these variations and thus may allow for improved diagnosis. For instance, FDG signal can appear to be lower than anticipated despite a large influx of neutrophils and macrophages observed [29]. This decrease in signal comes from an increase of the air fraction area in COPD lungs and is integrated in full lung area quantification methods [30]. Because of these variations, quantitative methods need to be applied regionally. To do so, tissue/cell specific radiotracers will need to be developed.

#### Single-photon emission computed tomography

SPECT imaging has been used to visualize and assess pulmonary airways. Planar scintigraphy of the lungs has been utilized to obtain information about ventilation and perfusion in patients with emphysema who have undergone lung volume reduction surgery. Three-dimensional ventilation/perfusion (v/q) SPECT imaging procedures have been used to help diagnosis [31]. COPD patients have also been evaluated using Technegas (^99m^Tc-labelled carbon particles). Technegas has the potential to provide valuable information for the diagnosis of COPD as it has been demonstrated to correlate positively with spirometric lung function. However, further studies are needed to validate the method.

**Fig. 4:**
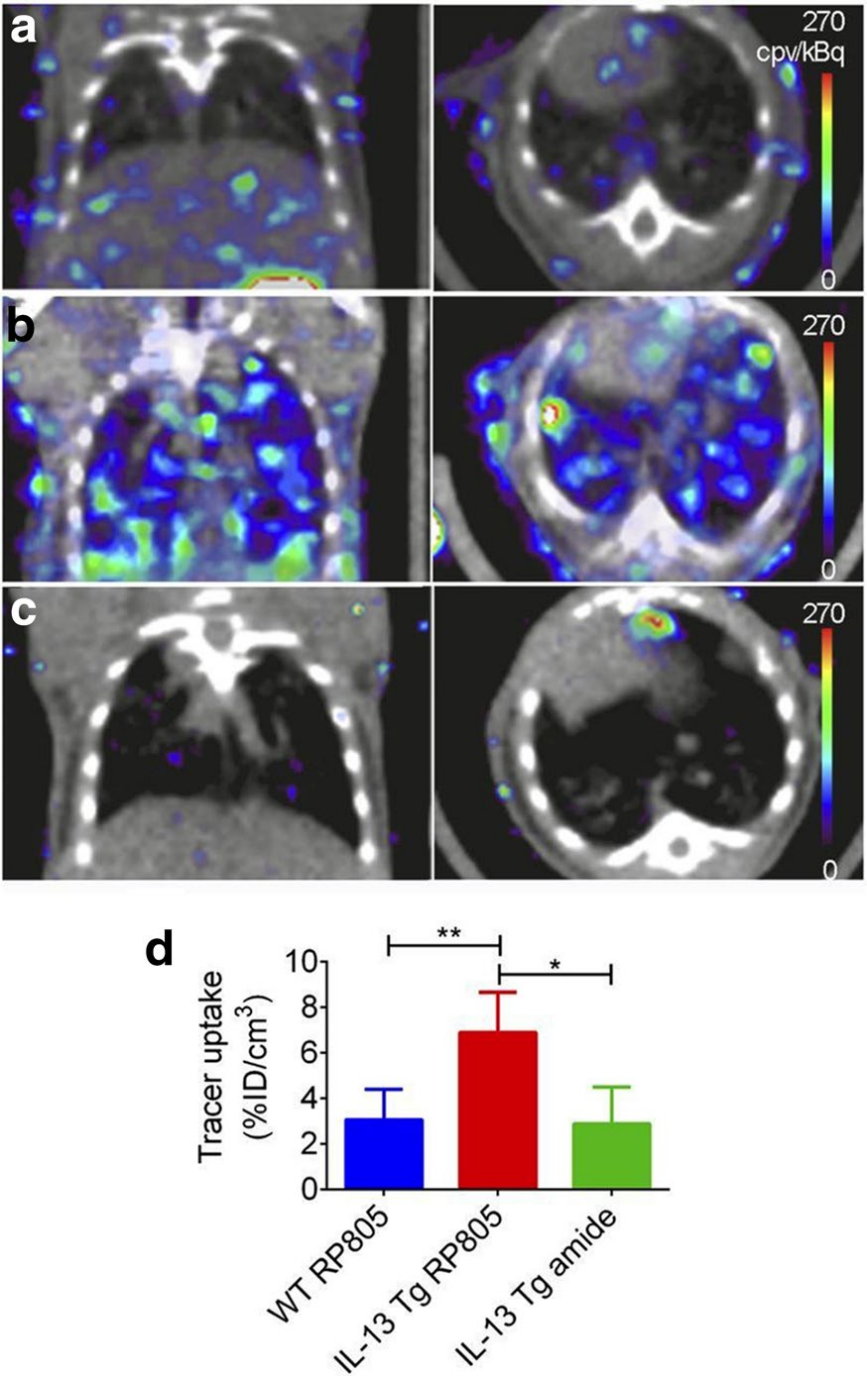
In vivo small-animal SPECT/CT imaging of MMP activation. **a**–**c** Examples of coronal (left) and transversal (right) views of fused small-animal SPECT/CT images of WT mice injected with RP805 (**a**) and CC10-IL-13 Tg mice injected with RP805 (**b**) or its control, amide analog tracer (**c**). **d** Small-animal SPECT–derived quantification tracer uptake in lungs. *N *= 5, 6, 13, and 5, respectively, for WT mice injected with RP805 and CC10-IL-13 Tg mice injected with RP805 or amide, control analog. *P, 0.01. **P, 0.001. cpv 5 counts per voxel; ID 5 injected dose. (This research was originally published in JNM [32]. © by the Society of Nuclear Medicine and Molecular Imaging, Inc.)

Macrophages play an important role in COPD, and the presence of activated macrophages is well established in the context of pulmonary inflammation in COPD. An early study used a radiolabeled version of PK11195, a translocator protein-targeted agent ^11^C-PK11195 in COPD, asthma and healthy patient cohorts [27]. Development of molecular imaging agents specific to matrix metalloproteases (MMPs) secreted by macrophages and other inflammatory cells would also aid in COPD diagnosis. MMPs play a key role in lung inflammation as they are responsible for the degradation of extracellular matrix as well as tissue remodeling [32, 33]. Several types of MMPs have been shown to be relevant as potential targets for COPD diagnosis. The role of MMP-9 and MMP-12 in COPD staging have generated enough interest for Kondo et al. [34] to evaluate four derivatives of ML5, a potent MMP inhibitor, as probes suitable for PET imaging. The fluorinated compound ^18^F-IPFP, resulting from the conjugation of a prosthetic group ^18^F-NFP with iodinated ML5, conserved high affinity after labeling and exhibited higher uptake in the lungs of a murine COPD model when compared with healthy murine lungs on in vivo images. Additionally, Golestani et al. [32] were able to successfully image MMPs by SPECT/CT using the ^99m^Tc-labelled macrocyclic MMP-targeted agent RP805. In a lung-specific IL-13 knockout mouse model, the accumulation of RP805 was 2.5-fold higher than in wild-type mice (Fig. 4). The radiotracer uptake correlated with MMP-12 content and also with CD68, a macrophage marker.

## Imaging modalities in prognosis

In recent years, it has become clear that pulmonary function testing does not correlate well with functional status of patients, anticipated decline in lung function over time, and mortality prediction. This shortcoming of PFT is reflected in the recent 2017 GOLD group classification system whereby escalation of medical therapy in the longitudinal management of COPD is now exclusively based upon the functional status of the patient and their exacerbation frequency rather than the prior spirometrically based classification system. For example, the 6 min walk has been an integral part of calculating long term mortality in the BODE scoring system [35, 36]. During the earlier days of GOLD staging Forced Expiratory Volume in one second (FEV1) was exclusively used as the classifying marker of pulmonary function [37]. However, limitation of using FEV1 as a marker of disease severity became apparent especially when clinically relevant outcomes such as frequency of exacerbation and patient symptomatology were considered. Based on these building experiences, BODE continues to be considered a strong predictor of survival, but the latest GOLD staging is now primarily focused on the frequency of COPD exacerbation and scoring for symptomatology such as the modified Medical Research Counsil Dyspnea Score (MMRC) and COPD Assessment Test (CAT) [38].

More recently, frequency of COPD exacerbation has become an important outcome with significant financial and clinical impact. Prediction and prevention of COPD exacerbations has become a priority in the effort to reduce morbidity and mortality and to control healthcare costs. Unfortunately, FEV1 has not been found to correlate well with frequency of exacerbations regardless of COPD phenotype [19]. In this environment, a number of studies have attempted to identify imaging methods useful in assessing future exacerbation risk. Two studies involving subjects enrolled in two major longitudinal, observational studies of COPD (COPDGene and ECLIPSE) identified bronchial wall thickness, extent of lung emphysema and pulmonary artery-to-aorta ratio as significant predictors of acute exacerbations of COPD. Quantitative analysis of CT images of 1002 patients with COPD enrolled in the COPDGene study reported that every 5% increase in emphysema extent (in patients with > 35% total emphysema) and every 1-mm increase in bronchial wall thickness (the mean value of six segmental airways) were associated with 1.18- and 1.84-fold increases in COPD exacerbation rate, respectively [3]. In another study of nearly 3500 from the COPDGene study, Wells et al. [39] identified a PA/Ao ratio of > 1 as significantly associated with a history of severe exacerbations, a finding that was validated in the ECLIPSE cohort where in a multivariate analysis a PA:A ratio of > 1 was associated with increased risk of severe COPD and exacerbations at 1 and 3 years (Odds Ratio (OR) 2.8 and 3.81 for severe exacerbations at year 1 and year 3, respectively; OR 2.17 and 6.68 for all exacerbations at year 1 and year 3, respectively).

Interestingly, in a separate analysis of 2138 patients enrolled in the ECLIPSE study, Hurst et al. [40] reported that while every 5% increase in extent of low-attenuation areas was a predictor of increased exacerbation occurrence at 1 year of follow-up, this association did not survive multivariate analysis. The findings of this study may be reconciled with the Han study, as it appears that bronchial wall thickness may have a greater influence on exacerbation rates in subjects with levels of emphysema < 35% [3].

Both quantitative and non-quantitative CT-based assessment of COPD have also been directly associated with mortality. For example, in the Norwegian GenKOLS study of 947 ever-smokers, Johannessen et al. [41] reported an increasing mortality associated with progressively higher rates of emphysema as assessed by the quantitative emphysema severity metric of percent low attenuation area inferior to − 950 HU. Interestingly, in an analysis of 3171 subjects enrolled in the COPDGene study, Lynch et al. [42] reported significant associations between mortality and emphysema classification as assessed by the five-point, non-quantitative Fleischner grading scale. Even after adjustment for the low attenuation area inferior to − 950 HU, non-quantitative emphysema severity was associated with increased hazard ratios for mortality in moderate and confluent emphysema grades [42].

Taken together, these studies suggest that use of CT may be able to stratify patients with COPD on the basis of prognosis both in terms of anticipated exacerbation and mortality rates. In turn, these populations may then be targeted for earlier, more aggressive medical therapy. Such imaging-based prognostication may urge the adoption of an early lung-transplantation referral strategy for certain high-risk groups.

## Imaging modalities in therapy selection

Although not entirely independent of prognosis, another clinically valuable application of imaging modalities in COPD is the identification of patient subpopulations/phenotypes most likely to benefit from selective, targeted therapies. The premier example of such an imaging application has been in the identification of patients who may benefit from surgical and endobronchial lung volume reduction strategies. In the landmark randomized trial comparing surgical lung volume reduction (LVRS) to medical therapy for severe emphysema in 1218 patients, overall mortality was not significantly different between the surgically and medically treated groups (0.11 death per person-years in both groups) [43]. However, a partially pre-specified subgroup analysis suggested that the distribution of emphysema as assessed by high-resolution CT was important in distinguishing patients that could be expected to benefit from LVRS from those that could be expected to experience harm [43]. In particular, patients with upper-lobe predominant emphysema and a low maximal workload post pulmonary rehabilitation were found to derive significant mortality benefit from LVRS (RR 0.47, P = 0.005) when compared with patients randomized to medical therapy. These patients also experienced improvement in quality of life and exercise capacity. On the other hand, patients with predominantly homogeneous emphysema and a high maximal workload were found to experience significant harm from the surgical intervention, with a statistically higher risk of death (RR 2.06, P = 0.02) when compared with the medical therapy group [43].

In a randomized trial investigating the benefit of a bronchoscopic approach to lung volume reduction with placement of one-way endobronchial valves, heterogeneity of emphysema as assessed by HRCT was used both as an enrollment as well as a targeting strategy. In this study design, the lobes targeted for endobronchial valve placement were required to have greater emphysematous involvement (as assessed by proportion of voxels inferior to − 910) than the ipsilateral, adjacent lobe meeting a pre-specified minimum threshold [44]. Improvements in lung function, exercise tolerance and symptoms were appreciated in the bronchoscopic intervention group of this trial. While the use of emphysema distribution and heterogeneity in these two trials may appear to be somewhat gross and imprecise, it does demonstrate that the adoption of medical imaging can be instrumental in patient selection for therapies in COPD.

Aside from these mechanical therapies for advanced emphysematous disease, the use of CT to monitor the response to medical therapies have also been investigated. Notably, in a study of 3661 COPD patients enrolled in the COPDGene study, Kim et al. [45] reported that airway wall thickness as assessed by multiple metrics including wall area percent, was found to be significantly increased in COPD patients that were responsive to bronchodilator therapy as defined by American Thoracic Society criteria.

Other examples of imaging modalities in evaluating responses to therapy are significantly more investigative but may serve as harbingers of future applications. In particular, the feasibility of quantifying the dynamic effects of bronchodilators in patients with COPD has been demonstrated both with serial HRCT as well as hyperpolarized ^3^He MR imaging [46, 47]. The latter is of particular interest as Kirby et al. reported significant improvements in ventilation (quantified as ventilation defect percent), which could be detected even in COPD subjects without significant bronchodilator response when evaluated by spirometry.

In a more recent study, Iyer et al. [48] demonstrated the feasibility of using dual-energy CT (DECT) to evaluate the presence of re-distribution of pulmonary blood flow in response to a vasodilator, sildenafil. In their investigation, 17 current smokers with normal pulmonary function as assessed by PFTs were divided into 2 groups based on CT evidence of centrilobular emphysema, where subjects with evidence of emphysema were considered susceptible-smokers (n = 10) and subjects without evidence of emphysema were considered non-susceptible smokers (n = 7). The authors reported that susceptible smokers were characterized by increased central pulmonary arterial volumes, believed to represent increased peripheral resistance, and that administration of sildenafil in these same subjects resulted in decreased heterogeneity of regional pulmonary perfused blood volume, a surrogate for pulmonary blood flow; an effect not appreciated in the non-susceptible smokers. Such findings suggest the presence of a reversible endothelial pathology in smokers with imaging evidence of emphysema.

Additionally, radiopaque noble gases like xenon have been used in combination with DECT to inform about ventilation and perfusion of the lungs. In this case, it is possible to discriminate xenon and other substances like calcium by their respective absorption properties at low and high energies. Recent work from Lee et al. [49] compared parenchymal attenuation changed in COPD patients. The cohort underwent xenon ventilation DECT during wash in and wash out phases and PFTs. The investigators found out that xenon ventilation change correlated with parenchymal change and predicted more accurate PFTs.

## Imaging modalities in clinical trials

Imaging modalities can facilitate the development of novel and investigative therapies for COPD by transforming the design of clinical trials in three significant ways: (1) identifying COPD patients at early preclinical stages that may be most responsive to disease-modifying medical and lifestyle interventions, (2) refining target COPD populations for specific therapies by employing imaging techniques described in previous sections and (3) furnishing sensitive study end-points that correspond to incremental therapeutic benefits not detectable by global functional assessment, thus allowing for parsimonious study design. In the previous section we described several imaging techniques that may be employed to enrich target populations for therapies including lung volume reduction, bronchodilators, and vasodilators. Such techniques may be used to target specific COPD phenotypes for intervention in future clinical trials.

Perhaps the most salient examples of this second use of imaging modalities in clinical trial design have been the studies of enzyme therapy in alpha-1 antitrypsin deficient patients. Investigators from the RAPID Trial Study group as well the Danish-Dutch and EXACTLE studies evaluating the efficacy of intravenous alpha-1 proteinase inhibitor augmentation in this patient population employed changes in CT-densitometry metrics as primary endpoints in these studies and reported statistically significant reductions in emphysema progression as assessed by these markers, efficacy which was not detectable by global FEV1 assessment over the 2–3 year courses of these trials [50, 51]. More recently, these methods were instantiated in the more generalizable population of the MESA Lung study. In this longitudinal cohort study, Aaron et al. [4] reported that regular aspirin use was associated with slower progression of emphysema as assessed by CT (percent emphysema, voxels inferior to − 950HU). These examples underscore the potential value of imaging modalities as surrogates for significant patient-centered outcomes not only in rare phenotypes of COPD but in COPD broadly where statistically significant changes in global lung function as well as patient-centered outcomes such as functional status and mortality may take years or decades to detect in prospectively designed trials.

## Conclusion

Despite medical tools currently available to clinicians, COPD is difficult to diagnose as symptoms are confounding with other pulmonary diseases. As inflammation is a major component of COPD along with intimate lung structure modifications, a cellular and molecular imaging approach is necessary to better understand, diagnose and treat the disease. Active research in the field is ongoing and will eventually permit monitoring of leukocyte infiltration and matrix degradation for a differential diagnosis of lung disease. Indeed, the combination of conventional imaging technique such CT and MRI with molecular imaging tools like PET and SPECT is anticipated to improve the field and to expand the possibilities to detect crucial elements in COPD pathogenesis. These emerging imaging technologies are anticipated to foster and promote novel understanding of pathogenesis, early detection for effective prevention, and rapid and timely development of potentially disease-modifying therapeutics.

## Abbreviations

COPD: chronic obstructive pulmonary disease
PFT: pulmonary function testing
CT: computed tomography
MRI: magnetic resonance imaging
SPECT: single photon emission computed tomography
PET: positrons emitting tomography
KeV: kilo electron volt
WA%: wall area percent
LAA%: low attenuation area percent
HU: Hounsfield unit
FEV1: force expiratory volume in the first second
MLD: mean lung density
RV: residual volume
TLC: total lung capacity
MDCT: multi detector computed tomography
PBF: percent body fat
DLCO: diffusion capacity of carbon monoxide
VDP: ventilation defect percentage
OEMRI: oxygen-enhanced magnetic resonance imaging
ADC: apparent diffusion coefficient
MR: magnetic resonance
FDG: fluoro deoxy glucose
MMP: matrix metalloprotease
OR: odds ratio
LVRS: lung volume reduction surgery
HRCT: high resolution computed tomography
DECT: dual energy computed tomography
